# Engagement in local and collaborative wildfire risk mitigation planning across the western U.S.—Evaluating participation and diversity in Community Wildfire Protection Plans

**DOI:** 10.1371/journal.pone.0263757

**Published:** 2022-02-09

**Authors:** Emily Palsa, Matt Bauer, Cody Evers, Matt Hamilton, Max Nielsen-Pincus

**Affiliations:** 1 School of Environment and Natural Resources, The Ohio State University, Columbus, Ohio, United States of America; 2 Department of Environmental Science and Management, Portland State University, Portland, Oregon, United States of America; 3 Sustainability Institute, The Ohio State University, Columbus, Ohio, United States of America; USDA Forest Service, UNITED STATES

## Abstract

Since their introduction two decades ago, Community Wildfire Protection Plans (CWPPs) have become a common planning tool for improving community preparedness and risk mitigation in fire-prone regions, and for strengthening coordination among federal and state land management agencies, local government, and residents. While CWPPs have been the focus of case studies, there are limited large-scale studies to understand the extent of, and factors responsible for, variation in stakeholder participation—a core element of the CWPP model. This article describes the scale and scope of participation in CWPPs across the western United States. We provide a detailed account of participants in over 1,000 CWPPs in 11 states and examine how levels of participation and stakeholder diversity vary as a function of factors related to planning process, planning context, and the broader geographic context in which plans were developed. We find that CWPPs vary substantially both by count and diversity of participants and that the former varies as a function of the geographic scale of the plan, while the latter varies largely as a function of the diversity of landowners within the jurisdiction. More than half of participants represented local interests, indicating a high degree of local engagement in hazard mitigation. Surprisingly, plan participation and diversity were unrelated to wildfire hazard. These findings suggest that CWPPs have been largely successful in their intent to engage diverse stakeholders in preparing for and mitigating wildfire risk, but that important challenges remain. We discuss the implications of this work and examine how the planning process and context for CWPPs may be changing.

## 1. Introduction

Models of collaborative planning have proliferated over the past several decades in the U.S., particularly in environmental and natural resource management contexts [1]. Extensive experimentation in models of collaborative, community-based, and network governance [2] are increasingly reflected in congressional, regulatory, and programmatic policies of the U.S. government [3, 4]. These policies encourage broad and diverse participation in policy, planning, and management decisions, particularly in settings where power is broadly distributed across multiple spatial/administrative levels and actions among individuals and organizations are highly interdependent.

Wildfire risk mitigation is an archetypal multilevel environmental governance challenge, featuring a tight coupling of human-environment interactions within and between community, landscape, and regional scales [5–7]. The potential transmission of wildfire across distinct management jurisdictions elevates the importance of coordination of risk mitigation measures among large and diverse groups of stakeholders who share ownership, management, or other responsibilities and interests for fire-prone landscapes [8, 9]. While the scale of the challenge of addressing wildfire risk has prompted significant attention and response at state and national levels, the past several decades have witnessed extensive local-level innovation as well, including initiatives such as the Firewise program, the Good Neighbor Authority program, and Community Wildfire Protection Plans (CWPPs) [10, 11].

CWPPs—the focus of this paper—are the most extensively used wildfire risk mitigation planning tool for wildland-urban interface communities in the U.S. and provide rich opportunities to examine collaborative environmental problem-solving in fragmented and decentralized risk governance systems [12]. Guidance for CWPP development is vague, which has spurred considerable diversity in approaches for plan development as well as the scope of risk mitigation priorities identified in plans themselves [13]. This variation notwithstanding, CWPPs do share certain features, including the identification of fuels reduction priorities, recommended actions for reducing structure ignitions, and the collaborative engagement of stakeholders in the planning process [14]. This emphasis on collaboration motivates our study, which evaluates participation and diversity in CWPP planning processes. Specifically, we draw upon a dataset of 1056 CWPPs from 11 states in the western U.S. We use rosters of participants in these plans to measure levels of participation and diversity in CWPPs, and we estimate models to improve understanding of the factors that shape these measures of collaborative risk mitigation planning.

We make several contributions to the fields of collaborative environmental decision-making and human dimensions of wildfire. Most generally, our analysis demonstrates the extensive scope of local wildfire risk mitigation planning processes across the western U.S., which includes thousands of stakeholders participating in thousands of plans that span neighborhoods to multiple counties in scale. We also document substantial variation in engagement in these planning processes and highlight factors that influence participation and stakeholder diversity. In so doing, we shed light on the social processes that precede the implementation of risk mitigation actions. Prior research on this subject has primarily relied on case studies, and our large-n analysis enables a much more extensive evaluation of a range of factors that shape social processes in local risk mitigation planning. In the following sections, we describe the CWPP model used for local and collaborative wildfire risk mitigation planning in the U.S. and explain why participation and diversity are important in local planning processes. After describing our data collection and analysis methodologies, we present our results, which reveal that participation and diversity vary significantly by state, spatial/administrative level of plans, and land ownership diversity within planning jurisdictions. We conclude by highlighting how our results advance theoretical understanding of local environmental planning in fragmented and decentralized landscapes, as well as the implications of our results for local planners and other risk mitigation stakeholders.

## 2. Participation and diversity in local collaborative wildfire risk mitigation planning

### 2.1 Community Wildfire Protection Plans: A model system for local collaborative risk governance

Introduced as part of the Healthy Forest Restoration Act (HFRA) of 2003, CWPPs sought greater involvement of local communities in wildfire risk mitigation and forest management [13]. The HFRA emphasized the importance of developing CWPPs in a collaborative manner involving a diverse set of participants, and would require approval from local government, local fire protection, and the state entity responsible for forestry. The act further directed federal agencies to collaborate with communities in identifying and prioritizing of areas needing hazardous fuels treatment and required that half of federal funds directed at fuels reduction be conducted within the wildland urban interface, including those specified within CWPPs. CWPPs subsequently grew to become the one of the primary tools for local risk mitigation planning, in part because plans provided access to state and federal grants for managing hazardous fuels. Over two decades, new and updated plans continue to be developed. This two decade period also coincides with growing wildfire risk throughout the western U.S. [15] and accompanying increases in resources allocated to wildfire prevention and response. For example, congressional appropriations for emergency fire suppression have risen from 0.92 billion USD annually in the 1990s to over 3 billion USD [16]. Another trend that has accompanied the development of CWPPs is the growing set of constraints on public land management agencies due to both declines in staff and the increasingly broad mandate for how public lands should be managed [1]. Against this backdrop, agencies have increased their reliance on non-governmental stakeholders, which provide both the physical capacity to accomplish work as well as a core vehicle for garnering public support and legitimacy [17].

While the stated objectives of CWPPs emphasize implementation of risk mitigation activities such as fuels reduction projects, prior research has documented the capacity of CWPPs to also foster the development of social capital, which can help stakeholders engage in collective action to mitigate risk. In particular, there is evidence that CWPPs promote cooperation and social learning among participants [11, 13, 18], which is crucial for grappling with complex environmental management challenges [19–21]. Local residents can also provide local knowledge, such as indigenous or anecdotal knowledge, that wildfire professionals require [2, 22]. Moreover, CWPPs may help to increase hazard awareness, and help local stakeholders generate consensus and understanding of mitigation actions and thus be more likely to engage in recommended behaviors [23]. Participation can also increase local buy-in among individuals wary of the intentions behind government intervention, as collaboration between government officials and community members during CWPP development may build trust between these groups [2]. Citizen participation in the planning process can also empower local residents to take greater responsibility for addressing wildfire risk [22].

While the HFRA emphasizes collaboration as a core feature of the CWPP process [24], the HFRA does not specifically define collaboration [13] outside of requiring the agreement of local and state governments and the consultation of federal agencies and other interested parties. Given the large number of CWPPs that have been developed, there is a need to take stock of how plans have engaged stakeholders and to develop insight into the factors that shape participation and diversity in the planning process.

### 2.2 Participation and diversity in environmental hazard planning

Community participation in planning is often necessary for addressing environmental problems owing to the complexity of the issues involved and the number of overlapping interests and concerns. In a general sense, broad participation in planning provides important information to public officials while increasing the likelihood that planning outcomes reflect the interests and concerns of diverse stakeholder groups. As a result, participation can improve the decisions that agencies and public officials make and create legitimacy and public buy-in. Involving diverse elements of the local community may reveal vulnerabilities or capacities that impact how communities are able to contribute to mitigation efforts and anticipate, respond to, and recover from natural hazards. Participation can facilitate implementation as planning efforts gradually become institutionalized within the community. It should be noted that some studies suggest that diverse participant groups may not be advantageous as increasingly diverse core values and beliefs can create unique challenges which may impact the quality of outputs [25]. Although increased participant group diversity can create challenges, this goal of the HFRA is supported by a broad body of literature indicating that synthesizing talents, knowledge, skills, and attitudes from a large, diverse group promotes more effective solutions and institutional innovation [1, 2].

**Fig 1:**
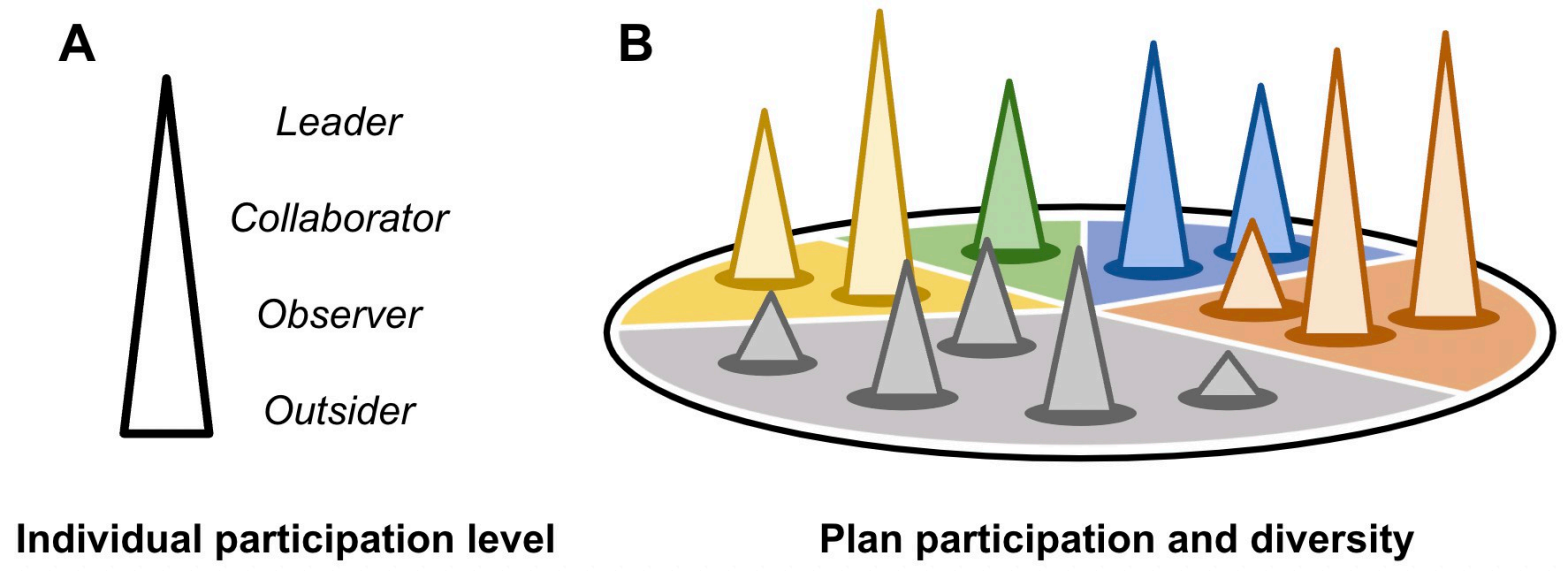
Collaborative planning as the combined result of the number and degree to which individuals participate in planning processes (Panel A), in addition to the groups, interests, and concerns that they represent (Panel B).

What constitutes meaningful participation in planning processes, however, is often unclear [26]. Engagement by individuals in planning is often conceptualized as a ladder [27], where the lowest rungs represent simple awareness while higher rungs involve occupying increasingly central roles in the planning process (Fig 1A). Through enhanced engagement, peripheral individuals may become more involved in the planning process through efforts that broaden the circle of engagement (i.e., including more outsiders in the plan) and increase engagement of those already within the circle (Fig 1B). In successful wildfire risk mitigation planning processes, a disengaged landowner with little knowledge of fire may become an active observer of efforts to reduce wildfire risk and may subsequently champion community-level risk mitigation actions, maintain involvement in this process over time, and may eventually take on leadership roles. Planning processes serve as a mechanism through which community engagement occurs [26]. Some planning processes are mere technocratic exercises that seek minimal external input; others may represent backroom power-brokering; others still emerge organically through grassroots-level mobilization. True collaborative planning engages core stakeholders as well as the diversity of other stakeholder groups [28]. Expanding engagement leads to increased participation and diversity, which constitutes the social capital upon which implementation of the plan often depends.

Prior research highlights a range of factors that affect participation and diversity in environmental planning. In the context of wildfire risk mitigation activities, the literature indicates participation is strongly predicted by biophysical risk including hazard levels and to a lesser extent housing structural density. Ojerio et al. [29] demonstrated that CWPPs were significantly more likely to be developed in more hazardous landscapes. Additionally, socially vulnerable communities (e.g., those living in poverty, or of vulnerable age) are less likely to participate in wildfire mitigation activities [29, 30]. Financial incentives can often have a significant impact on plan development and stakeholder participation due to the disparity of economic and planning means among communities [29]. Literature from areas outside of environmental planning provides insight into predictors of environmental planning participation. In the context of health-related support groups, there are many factors that predict participation, including the convenience and flexibility of the opportunities for participation, and the individual’s perceived credibility [31]. Perhaps these findings from the health fields hold some merit in the context of environmental planning as the perceived credibility of the organization orchestrating public meetings and the convenience of those meetings for the community members may influence participation. Existing literature indicates private consultants, used in many CWPP processes, may diminish collaboration [32]. Conversely, it is possible that the diversity of land ownership within the planning area may result in a more diverse planning group as planners and community members seek participation from all land ownerships. Nielsen-Pincus et al. [33] demonstrated that communities with more diverse land ownerships experienced increased predicted risk to local housing, highlighting the need for greater diversity in participation, especially in communities with lower levels of social capital. A larger population base and more urban communities may also result in higher levels of participation and diversity of participants as there is a denser pool of affected stakeholders. Other planning process and planning context factors may also influence these metrics, such as the state in which it is developed, the year the CWPP was published, the spatial or administrative level at which the CWPP is developed, or if the CWPP is new or an update of a preexisting plan.

## 3. Methods

We analyzed CWPPs from 11 states in the western U.S. that were drafted between 2001 and 2021. We measured participation and diversity among plans, which we defined as the total count of individuals who contributed to the development of plans and the diversity of affiliations of these individuals, respectively. Finally, we examined how levels of participation and stakeholder diversity vary as a function of factors related to planning process, planning context, and the broader geographic context in which plans were developed.

### 3.1 Identifying plans

We used a three-step process to identify CWPPs. We first identified online databases of plans for each state and downloaded all CWPPs provided on these websites. We then used targeted keyword searches, such as the name of a county, state, or sometimes community followed by “CWPP”, or “Community Wildfire Protection Plan” to identify individual plans not listed on state websites. In addition, we searched the plans themselves for both prior versions of that particular plan, as well as references to other plans that were used as models. Sources of CWPPs varied substantially by state. Some states had a comprehensive repository of CWPPs (e.g., Colorado). Other states had no comprehensive publicly available records, and in these cases, we used keyword searches, we reviewed state and municipal agency websites, and we reached out to key practitioners to cross-validate our records and to identify missing plans. All plans were downloaded and renamed by state, plan name, and year published. For each plan, we recorded the plan name, creation date, state/county affiliation, and classified each plan by its spatial/administrative level: county, fire protection district, or community. A fire protection district is a legal boundary in which a fire authority provides fire response services. Of the three levels, communities encompassed the broadest spectrum of plans, including cities, subdivisions, and local neighborhoods. For plans that had been updated, we recorded each plan separately.

### 3.2 Mapping plan jurisdictions

We mapped CWPP planning jurisdictions for each plan identified above. When possible, we used spatial data directly associated with the plan (e.g., shapefiles) or that had been previously assembled (see S2 File for full records of data sources). For example, jurisdictional boundaries in Oregon were obtained from the Oregon Spatial Data Library [34]. In cases in which secondary data were not available, we relied on the maps included in most CWPP documents. Commonly, plan boundaries were clearly identified in map legends. We georeferenced these maps in Google Earth Pro. For CWPPs that lacked maps or clearly marked jurisdictional boundaries, we used written descriptions of the CWPP boundaries (e.g., references to surrounding highways or other landmarks), which were typically provided in introductory sections of plans. If no description was available, we used census-designated place boundaries to specify the boundaries of the CWPP based on the jurisdiction(s) described in the plan. Once mapped, we combined CWPP boundaries into a single spatial archive.

### 3.3 Identifying plan participants

**Fig 2:**
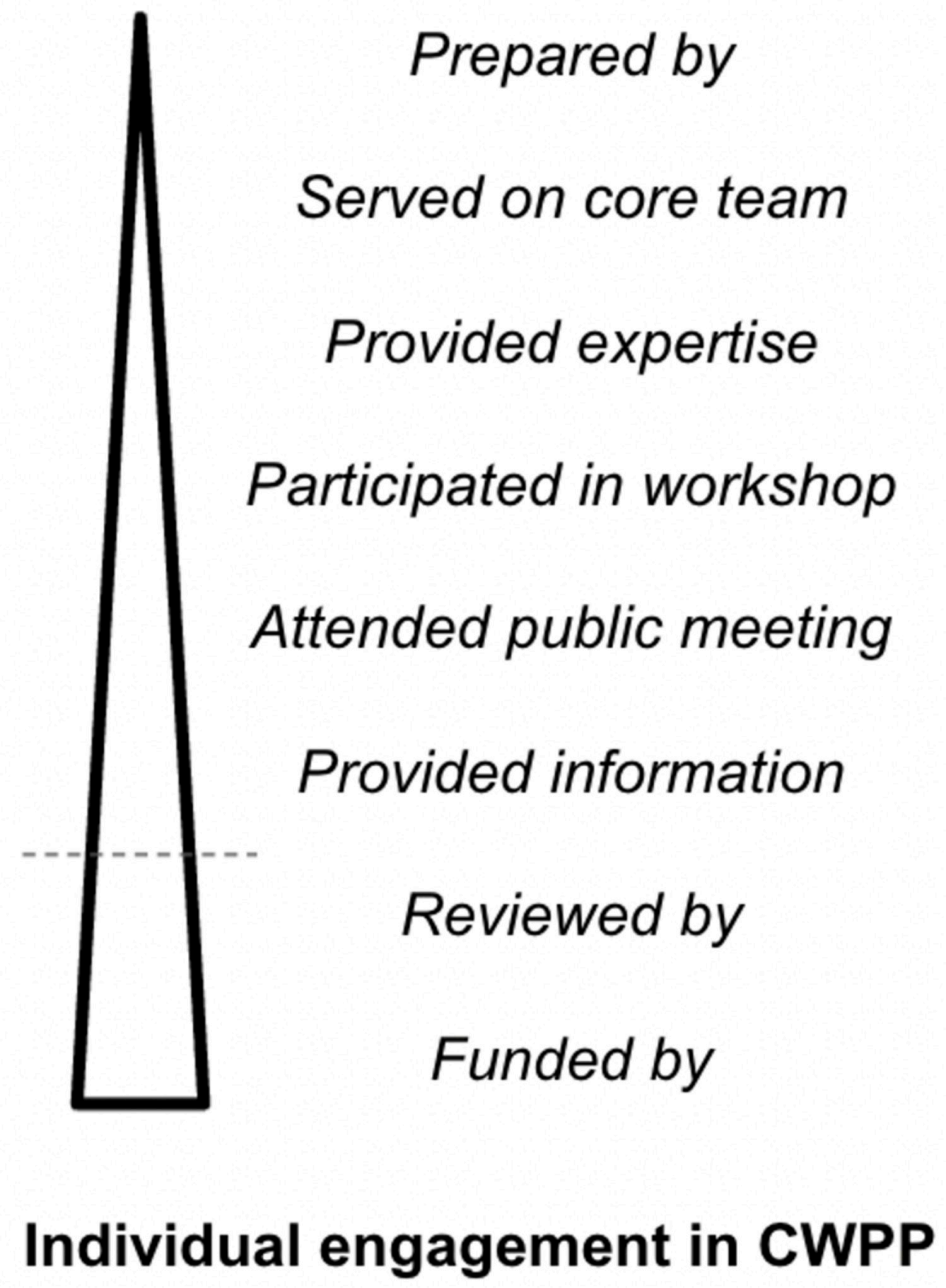
Common indicators of engagement by individuals within the CWPP. The dashed line represents the threshold of an individual engaging sufficiently to be considered a participant. Individuals above this line were considered participants in the CWPP and are the focus of this article.

We collected data on each plan’s participants and their respective organizational affiliations. To do so, we reviewed the entirety of each CWPP document for references to participation. Along with the full names and organizational affiliations, we recorded verbatim the statement(s) describing each individual’s contributions to the plan. These statements were used to determine whether individuals were sufficiently involved in plan development to be considered “participants”, rather than simply a funder, reviewer, or signatory (See Fig 2). For example, if a CWPP reported that an individual was “invited to the planning team” but provided no evidence that the individual attended meetings or otherwise contributed to the development of the plan, the individual was not included as a participant. Some CWPP documents did not list names of participants, but instead listed individuals’ organizational affiliations. In these cases, we documented the organization along with a missing name placeholder. Several plans included no information on participants and were documented as such. We standardized names of individuals to ensure consistent spelling and format (Last, First) throughout the dataset. For example, if the same individual was cited for participation with their nickname in one plan and another with their full name, each mention of that individual was changed to use their full name.

We characterized individuals based on their organizational affiliation type. Most individuals had a clearly stated organization for which they worked. Once we had a comprehensive list of all participant organizations, we standardized the organization names manually in a similar manner to that for individual names. Organizations were grouped into 17 affiliation categories (see S1 File for details).

### 3.4 Predicting plan participation and diversity

We created two regression models to gain insight into predictors of plan participation and diversity. Both dependent variables were fitted as a random-intercept mixed-effects model using the R package ‘lme4’ [35]. Our first dependent variable, participation, was derived from the participant count of each plan. Our second dependent variable, diversity, was calculated as the diversity of organizational affiliations of plan participants. We used the Shannon-Wiener Diversity Index to measure diversity [36]. Given that many plans represented updates, we modeled participation and diversity as random-intercept models, with the plan jurisdiction set to the grouping variable. Independent variables were screened for multicollinearity. All variables, including dependent variables, were standardized from 0 and 1 to aid in interpretation.

Independent variables used in the model related to the planning process, planning context, and the broader context in which plans were developed. *Private consultant* was a binary variable with a value of 1 if the CWPP was prepared by a private consulting firm and 0 otherwise. *CWPP revision*, a binary variable, measured whether the plan was an update of a prior plan developed in the same jurisdiction. *Planning level* was a categorical variable that indicated whether plans were developed at the community, fire protection district, or county level (the reference category). *Planning boundary area* measured the size of the jurisdiction (originally in square km but rescaled from 0–1 in the model). *Year published* was a categorical variable that divided the 2001–2021 period of analysis into three equal periods of time: 2001–2007 (the reference category), 2008–2014, and 2015–2021. The first period roughly corresponds to the initial pulse of CWPP development immediately surrounding the HFRA. *State* indicated the state in which plans were developed. *Population* measured the estimated population within the CWPP jurisdiction, based on data from the 2019 American Community Survey that were obtained from the National Historical Geographic Information System [37]. *Urban-rural gradient* measured the proportion of the population living in urban areas, using data from the U.S. Census that we obtained from the National Historical Geographic Information System [37]. Land ownership diversity was calculated from data obtained from the USGS Protected Areas Database [38], which we grouped into 14 major land classes. Using the proportions of areas in these classes we used the Shannon-Wiener diversity index (SWDI) to calculate diversity of land ownership, which we subsequently rescaled from 0–1. *Wildfire risk* was derived from the Wildfire Hazard Potential WHP dataset [39], which assigns pixels a ranking based on the potential for fires that would be difficult to suppress. Pixel values within CWPP jurisdictions were averaged. *Structural density* measured the number of housing units within each CWPP jurisdiction divided by the area of the jurisdiction. Data on housing units from the 2019 American Community Survey were obtained from the National Historical Geographic Information System [37]. *Social vulnerability index*, measured for each CWPP jurisdiction, was derived from the CDC/ATSDR Social Vulnerability Index [40].

## 4. Results

### 4.1 Characterization of Community Wildlife Protection Plans

We identified 1056 CWPPs in the western U.S. developed between 2001 and 2021 (Table 1). Nearly half of these CWPPs were developed in Colorado (259 plans) and California (249 plans). The spatial/administrative level at which plans were developed varied substantially among states. County- and community-level plans were the most common, with very few states having plans developed at the fire protection district level. Colorado differed notably in this respect, with 24% of plans developed at the fire district level. In most states, approximately half of the plans were prepared by private consultants. A private consultant prepared all of Nevada’s CWPPs, contrasted with only 9% consultant-prepared plans in Utah. Across all states, an average of 19 individuals participated in each CWPP, for a total of nearly 20,000 instances of participation. CWPPs developed in Idaho and Washington had the highest average participation rates while CWPPs in Utah and Wyoming had the lowest. While nearly all CWPP documents included records of participants (e.g., full names of individuals and/or organization names), the omission of this information varied from state to state. For example, approximately 10% of plans developed in Utah lacked information on participants.

**Table 1:** Characteristics of plans.

State	Total plans (n = 1056)	Spatial/administrative level (%):			Created by consultant (%)	Average participants per plan	No participant data (%)
		County	Community	Fire district			
AZ	44	38.6	61.4	0.0	50.0	19.8	0.0
CA	249	31.3	59.8	8.8	27.7	19.2	1.2
CO	259	19.3	56.4	24.3	39.4	14.3	3.9
ID	110	97.3	0.9	1.8	51.8	27.6	3.6
MT	53	98.1	1.9	0.0	64.2	18.5	0.0
NM	61	54.1	45.9	0.0	45.9	21.0	0.0
NV	37	86.5	8.1	5.4	100.0	19.8	0.0
OR	72	62.5	31.9	5.6	36.1	18.1	0.0
UT	66	12.1	87.9	0.0	9.1	9.2	10.6
WA	62	35.5	64.5	0.0	45.2	24.8	0.0
WY	43	88.4	11.6	0.0	51.2	14.2	2.3
ALL	1056	45.6	45.6	8.8	40.9	19.0	2.4

**Fig 3:**
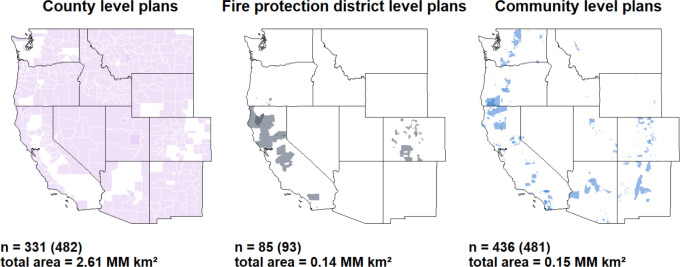
CWPP jurisdictions at three levels: County, fire districts, and community. Jurisdictional counts are shown below each map (with plan counts in parentheses), in addition to the jurisdictional area covered.

As noted in Table 1, a key source of variability across the dataset is the spatial/administrative level at which plans were developed. The relative prominence of plans at different levels likewise varied geographically as well (Fig 3). County-level plans represented nearly 90% of the total jurisdictional area covered by CWPPs, even though community-level plans were more numerous. The majority of fire protection district-level plans were developed in California and Colorado. Although community-level plans were developed in every state, these plans were clustered in regions with denser populations (e.g., in the Colorado Front Range region and throughout California). While county- and fire protection district- level plans adopted the jurisdictions of the administrative units in which they were developed, participants in community-level plans determined their own planning jurisdictions, which varied from 0.27 km^2^ to 21,123 km^2^.

**Fig 4:**
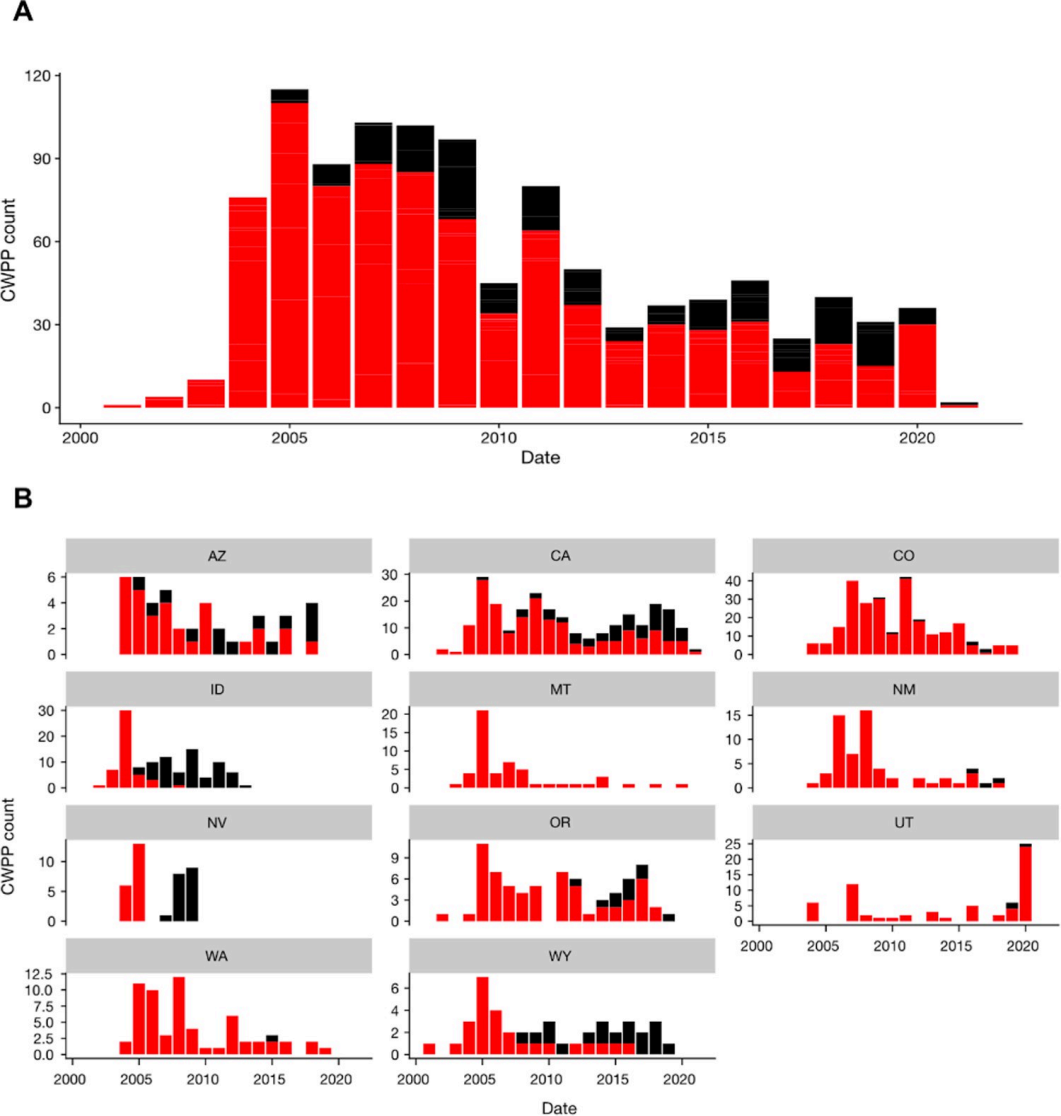
Development of CWPPs over time across all 11 states (panel A) and within each state (panel B). Plots distinguish between the creation of new plans (red) and revised or updated plans (black).

Plan development over time varied significantly from state to state (Fig 4). Generally, a pulse of CWPPs were developed during the mid-2000s, following the 2003 passage of the HFRA. Plan revisions were common in about half of the states to varying degrees. Some states published more than 40 plans in a given year, and others reached a maximum of six. Many states exhibited peak plan creation around 2005, and some had a second peak between 2010–2015. Plans in Utah did not peak until 2020. Following these peaks, some states’ development tapered off gradually (e.g., Washington) while others dropped off more substantially (e.g., Nevada).

### 4.2 Characterization of plan participants

We recorded 19,449 instances of individuals participating in CWPP development between 2001 and 2021, representing local, state, federal and tribal government, and a range of non-governmental and private sector organizations (Table 2). Individuals associated with local government or non-governmental organizations were the most common, defining 70% of all participants. Local fire districts or departments and county organizations were the most numerous affiliation groups, followed by unaffiliated individuals (e.g., a resident or community member), private businesses, and state departments of forestry and natural resources. Federal and tribal actors represented 15% of all participants, of which employees of the USFS and BLM were the majority. Tribal government representatives comprised only 0.6% of this category. State agencies made up the remaining 10% of CWPP participant groups.

**Table 2:** Organizational diversity in CWPPs based on affiliation group (n = 19,449).

Affiliation	Count	Percent
Federal & Tribal government (n = 3006; 15%)		
United States Forest Service	1344	6.9%
Bureau of Land Management	962	4.9%
Other federal agency	576	3.0%
Tribal government	124	0.6%
State government (n = 1944; 10%)		
State department of forestry or natural resources	1462	7.5%
Other state agency	482	2.5%
Local government (n = 7256; 37%)		
City organization	959	4.9%
County organization	2289	11.8%
Local fire district or department	3543	18.2%
Other local or regional government organization	465	2.4%
Non-governmental (n = 6296; 33%)		
Community fire organization	1133	5.8%
Homeowners association	383	2.0%
Individual	1918	9.9%
Non-profit organization	880	4.5%
Private business	1749	9.0%
University	233	1.2%
Unknown affiliation (n = 947; 5%)		

**Fig 5:**
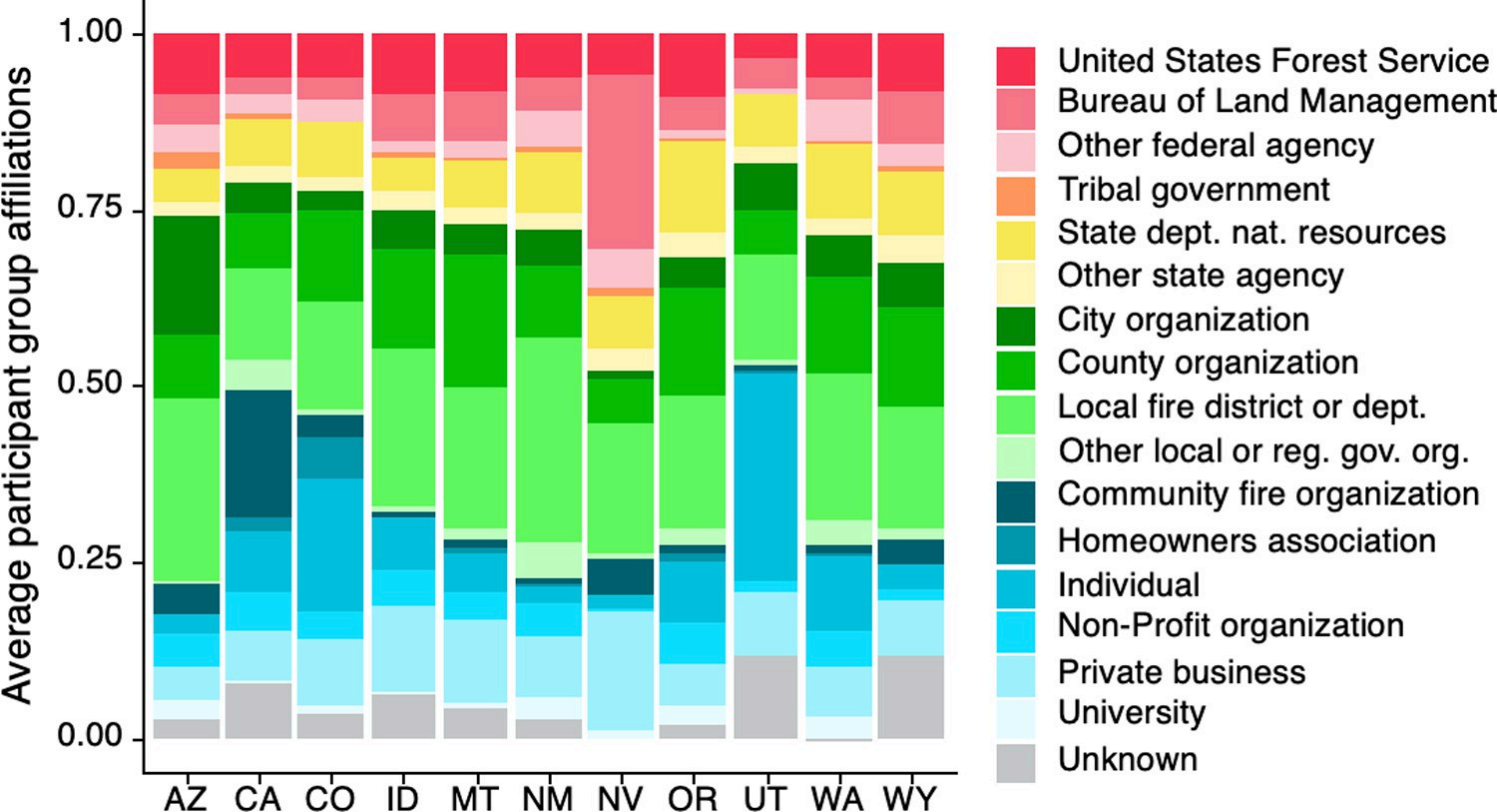
The average proportion of organizational diversity from CWPP participants by state. Color hues indicate high-level groupings of affiliation categories: red/orange, federal and tribal governmental agencies; yellow, state governmental agencies; green, local governmental agencies; blue, non-governmental organizations; grey, unknown.

Diversity among participant groups varied by state (Fig 5). For example, the representation of non-governmental organizations in Utah and California was relatively high. In Utah, non-governmental representation was primarily made up of individuals, and in California, primarily community fire organizations. In most states, approximately one-quarter of participants were representatives of state or federal governmental agencies. An exception is Nevada, in which patterns of participation reflected a more top-down model of planning in which state and federal agencies accounted for approximately one-half of participants.

### 4.3 Factors that affect participation and diversity in CWPPs

The mean participation count among plans was 19 and participation by plan varied between 1 and more than 200. Most plans had 14 or fewer participants, while plans with more than 50 were relatively few (6%). Plan diversity, calculated as SWDI, varied between 0 and 2.5 with a mean of 1.5. Values were slightly right-skewed, with a slight bimodal distribution resulting from the 46 plans with a diversity score of 0. Random-intercept models were fit to the data, first to the participation count by plan alone, and second to the diversity using participation counts as one of the independent variables (Table 3). In each model, the intercept was assumed to vary randomly by plan in order to account for the fact that approximately 20% of plans represented updates to previous versions. All variables were scaled to 0 and 1 meaning that model coefficients represent the percent increase in participation or diversity of the maximum value of each predictor compared to its minimum.

**Table 3:** Predictors of participation and diversity in CWPPs.

	Parameter	Participation	Diversity
	(Intercept)	0.09 (0.02) ***	0.49 (0.04) ***
Planning process	Participation		0.63 (0.07) ***
	Private consultant	0.01 (0.01)	0.03 (0.01) +
	CWPP revision	-0.03 (0.01) ***	0.04 (0.02) *
Planning context	Planning level: community	-0.03 (0.01) **	-0.03 (0.02)
	Planning level: fire protection district	-0.03 (0.01) **	-0.03 (0.03)
	Planning boundary area	0.09 (0.04) *	-0.01 (0.09)
	Year published: 2015–2021	0.01 (0.01)	-0.01 (0.02)
	Year published: 2008–2014	0.00 (0.01)	0.02 (0.02)
State	Arizona	0.00 (0.01)	0.06 (0.03) +
	California	0.02 (0.01) *	0.05 (0.02) *
	Colorado	-0.00 (0.01)	0.02 (0.02)
	Idaho	0.06 (0.01) ***	-0.05 (0.03) +
	Montana	-0.02 (0.01) +	-0.07 (0.03) *
	New Mexico	0.01 (0.01)	0.03 (0.03)
	Nevada	-0.02 (0.01) +	0.02 (0.04)
	Oregon	-0.00 (0.01)	0.02 (0.02)
	Utah	-0.03 (0.01) *	-0.12 (0.03) ***
	Washington	0.03 (0.01) **	0.08 (0.02) ***
	Wyoming^	-0.04 (0.01) **	-0.03 (0.03)
Geographic context	Population	0.03 (0.04)	-0.03 (0.09)
	Urban-rural gradient	-0.00 (0.01)	-0.02 (0.02)
	Land ownership diversity	0.03 (0.02) *	0.12 (0.04) **
	Wildfire risk	-0.01 (0.02)	-0.01 (0.04)
	Structural density	-0.05 (0.04)	-0.09 (0.09)
	Social vulnerability index	-0.02 (0.01)	0.02 (0.03)
	AIC	-1872.49	-286.92
	BIC	-1745.15	-154.68
	Log Likelihood	962.25	170.46
	p-value	8.9e-18	6.3e-31
	Num. obs.	990	990
	Num. groups	802	802
	Pseudo-R2	0.14	0.20

Notes

*** = p < 0.001

** = p < 0.01

* = p < 0.05

+ = p < 0.1

^ = A separate refactored model was used to estimate the effect of the withheld level (i.e., Wyoming).

Standard errors and statistical significance are noted for each predictor. Note that participation is used as a dependent variable in the left model and as an independent control in the right model.

Among factors related to the CWPP planning process, we found that participation levels were lower in CWPPs undergoing revisions. A number of factors related to the broader CWPP planning process affected participation. In particular, community- and fire protection district-level plans tended to have fewer participants than did county-level plans (the reference category). We also found that participation was greater in CWPPs with larger planning boundary areas. Participation levels varied considerably by state, and tended to be higher in California, Idaho, and Washington, and lower in Montana, Nevada, Utah, and Wyoming. Of all factors related to the broader geographical context in which plans were developed, only land ownership diversity affected participation, which tended to be higher in plans whose jurisdictions encompassed a more diverse set of ownership types.

Turning to our model of diversity, we found that the variation of stakeholders involved in CWPP development increased with the number of participants and was higher in CWPPs prepared by private consultants as well as in CWPPs undergoing a revision. Neither the spatial/administrative level at which CWPPs were developed nor the size of their planning jurisdictions affected diversity. We found that diversity was higher in CWPPs in Arizona, California, and Washington, and was lower in Idaho, Montana, and Utah. As in our model of participation, land ownership diversity was a positive predictor of participant diversity.

Neither participation nor diversity was associated with the timeframe the plans were produced, nor were they associated with the geographic context related to population, urbanization, structural density, wildfire risk, or social vulnerability.

## 5. Discussion

The comprehensive dataset of CWPPs presented in this paper describes patterns of stakeholder participation in local risk mitigation planning across the western U.S. between 2001 and 2021. Taken together, our results depict a dynamic bottom-up and local response to wildfire risk that the HFRA and other federal and state policies were intended to motivate. CWPPs cover the vast majority of the land base of the fire-prone western U.S. and have engaged large numbers of people with diverse organizational affiliations as active participants in wildfire risk mitigation planning. Such patterns of participation and diversity are noteworthy in the context of wildfire risk governance, which has traditionally adopted hierarchical and top-down models characteristic of other disaster risk reduction decision-making processes and most commonly seen in the incident command and wildfire response systems. CWPP participant groups are generally large groups of diversely affiliated participants. These groups are highly variable and influenced by a range of factors related to the CWPP planning process, the planning context, and the socio-environmental context in which plans were developed.

### 5.1 Contributions to scholarship

We found that characteristics of planning processes themselves were strong predictors of their levels of participation and diversity. In particular, our finding that larger groups are more diverse is consistent with earlier qualitative research on CWPPs [18]. In multi-stakeholder decision-making settings, participant diversity enables the combination of multiple sets of knowledge, which can help stakeholders better grapple with uncertainty and complexity [41]. To the extent that stakeholder diversity fosters learning [18], our results suggest that efforts to recruit greater numbers of participants may help CWPP planners craft more novel and adaptive risk mitigation plans.

Diversity was also higher among CWPPs prepared by private consultants. This finding contributes to active literature on the role of consultants in collaborative environmental planning processes, including CWPPs, which has highlighted potentially positive contributions of consultants (e.g., increasing the comprehensiveness of plan content [14]) along with undesirable effects (e.g., enabling local elites to control the planning outcomes [42]). One possible explanation of our finding is that consultants not only contributed to the scope of risk mitigation planning but also to the planning process itself, through outreach to groups that may not have otherwise participated.

Like many environmental planning processes, the CWPP model encourages adaptation of the scope and goals of plans over time, and a substantial portion of CWPP jurisdictions have developed one or more revisions of the initial plan. Although we found that participation declines in CWPP updates, we also found that diversity increases as plans are revised. Because our measure of diversity accounts for the proportional sizes of groups rather than raw counts of groups themselves, these results suggest that certain types of stakeholders may be overrepresented in initial CWPPs, relative to revisions, and that when smaller groups gather to revise a CWPP, they tend to retain representatives of the original set of stakeholders.

Our findings also contribute to scholarship on how institutions (in our case, CWPP planning processes) “fit” the socio-environmental systems in which they are embedded [43–45]. In particular, we found that representativeness is higher in CWPPs whose jurisdictions encompass a greater diversity of land ownership, suggesting that CWPP participants collectively reflect the range of likely constituencies for risk mitigation within plan boundaries. Scholarship on institutional fitness argues that such alignment is desirable because it signals greater responsiveness to diverse values and local variation in environmental management challenges [46, 47].

Risk-related variables—wildfire risk, structural density, population, vulnerability—did not predict participation or diversity in CWPPs. From a theoretical standpoint, this general finding is noteworthy because prior work has shown that local stakeholders can perceive risk and are cognizant of risk factors and that such awareness can spur collective action to mitigate risk [9, 48]. Our analysis suggests that hazard conditions and other factors that contribute to risk do not spur greater participation or more diverse participation in CWPPs, at least relative to factors included in the model. Likewise, the non-significant effects of risk-related variables highlight the possibility that more circumstantial factors (e.g., the availability of funding opportunities, prior involvement in other CWPPs) may influence stakeholder engagement more than recognition of the need for risk mitigation of the specific level of risk faced by a community.

### 5.2 Implications for management

CWPPs are representative of a diverse set of models for collaborative planning and multi-stakeholder decision-making processes that have taken root over the last several decades [6, 45, 49]. In the case of CWPPs, collaboration was mandated—however vaguely—in the Healthy Forests Restoration Act, which established guidelines for CWPP development [18]. However, the extensive variation in levels of participation and diversity of different stakeholder groups highlights the important influence of bottom-up factors. Understanding how these factors affect CWPP engagement points to specific opportunities for policy-makers and risk mitigation practitioners to increase local buy-in, improve responsiveness to diverse constituencies, and otherwise mitigate wildfire risk more effectively.

Our analysis reveals robust engagement of local stakeholders in wildfire risk mitigation planning. Nearly 40% of participants represent local governmental agencies and departments, while representatives of community fire organizations and homeowners’ associations as well as local residents account for an additional 17% of participants. The high level and diversity of local participants suggests that the CWPP model has been generally successful in engaging local stakeholders in wildfire risk mitigation planning. Likewise, these results suggest that federal and state agencies, which historically assumed both the responsibility and authority for wildfire risk management, have delegated some portion of these roles to local partners. Importantly, federal and state agency representatives constitute approximately 25% of participants, and this substantial level of engagement highlights the potential for meaningful collaboration among stakeholders with complementary skill sets, capacities, and resources. Participation in CWPPs is at least partially motivated by plan requirements and may be strongly motivated by desire to access or influence federal and states funds. Maintaining engagement is likely to vary across jurisdiction and state. These factors and others likely explain why diversity varied considerably between states. For instance, California plans involve a large proportion of community organizations, including the Fire Safe Councils, which commonly lead CWPP development in the state. Conversely, all of Nevada’s plans were led by private consultants and more than half were developed at the county level, indicating a more top-down approach that emphasized existing jurisdictional boundaries (i.e., counties), which may not necessarily align with the spatial scales of wildfire risk.

Our analysis likewise reveals opportunities to improve participation and diversity in CWPPs. In particular, our study highlights the need for more meaningful engagement of stakeholder groups that have historically been underrepresented in land use planning, such as tribal governments, whose participation in CWPPs was rare relative to other groups. More meaningful involvement of tribal governmental representatives in CWPPs could spur advances in community resilience, considering increased influence of tribal governments and associated organizations on land management as well as growing recognition of how indigenous fire practices maintained landscapes throughout the western U.S. [50]. Greater participation of tribal governments is especially important in regions with extensive tribal lands. For example, 28% of Arizona is tribal land, but only 0.24% of participants represented tribal governments in CWPPs developed in the state. This example also illustrates how representation may guide efforts to improve diversity within CWPP planning teams and the need for CWPP planners to assemble planning teams whose compositions are reflective of the sets of stakeholders within broader planning jurisdictions. Likewise, planning teams can anticipate and mitigate some of the pitfalls in planning processes that can emerge, such as representation gaps created when various groups frame the same problem in different ways [51].

Given the high proportion of forested land in the western U.S. that is managed by federal agencies, it is not surprising that certain CWPPs engage high numbers of federal and state agency representatives and low numbers of community members. Such patterns of diversity deviate from the spirit of the HFRA. With any community-based hazard planning, it is imperative that diverse local stakeholder groups are involved because they are most directly affected by hazard events and their support for disaster preparedness is crucial for large-scale risk mitigation efforts [52, 53].

### 5.3 Limitations and directions for future research

Planning documents are imperfect records of public participation, and the implications of increased participation are nuanced. While we distinguished between individuals merely named in the plan from those that actively participated based on language included in the document (see Fig 2), this dichotomy does not capture substantial differences in involvement and power among plan participants; it is possible that groups with high diversity by virtue of participant affiliations are nevertheless homogeneous in terms of the dominance of a subset of actors. Furthermore, while increasing participation in planning processes can contribute to trust and social capacity, involving a greater diversity of interests can lead to brokered influence peddling or fragmented social coalitions [26].

Future research on CWPP planning processes could seek to combine research approaches to better differentiate among levels of participation within the planning processes, in addition to tracking downstream impacts of plan diversity on project implementation and future planning processes. Through coding of planned and recommended risk mitigation actions outlined in CWPP documents, subsequent studies could evaluate the factors that shape the scale, scope, innovativeness, or other features of risk mitigation plans. More generally, content analysis of the corpus of plan texts could offer opportunities to assess how patterns of participation shape the sets of values that motivate priorities for risk mitigation actions, the quality of plans (e.g., the degree to which plans exceed minimal requirements), and ultimately the scope and scale of risk mitigation actions implemented within CWPP jurisdictions.

## 6. Conclusion

Community Wildfire Protection Plans are collaborative frameworks that outline local priorities for wildfire risk mitigation. Since CWPPs were introduced in the Healthy Forests Restoration Act two decades ago, thousands of plans have been developed across fire-prone regions of the U.S. Guidance for plan development is relatively vague, which has spurred considerable variation in the scope, scale, and goals of these planning processes. The large number and diversity of plans, as well as the substantial heterogeneity of socio-environmental contexts in which plans were developed, offer valuable opportunities to evaluate factors that shape participation and diversity in local and collaborative risk mitigation planning.

We leveraged a dataset of nearly 20,000 records of individuals’ participation in over 1,000 CWPPs in 11 states of the U.S. West, as well as attributes of individuals and plans to assess patterns of participation and diversity in CWPPs. Our results reveal a dynamic bottom-up response to wildfire risk that spans the vast majority of the land base of the fire-prone western U.S. and involves representatives of diverse organizational affiliations as active participants in wildfire risk mitigation planning. Against this backdrop, we likewise observe substantial variation in the scale of participation and scope of diversity of stakeholder groups in planning processes. We find that CWPP participation and diversity varies as a function of factors related to the CWPP planning process (e.g., the involvement of private consultants), the planning context (e.g., jurisdiction size), and the broader socio-environmental context in which CWPPs were developed (e.g., diversity of land ownership). Taken together, these results not only extend scholarly understanding of collaborative local risk governance in complex fragmented landscapes but reveal opportunities for managers to improve participation and diversity in planning processes.

## Supporting information

S1 File (DOCX)

S2 File (CSV)

S3 File (CSV)

S4 File (ZIP)

S5 File (R)

S1 Dataset (CSV)
