# Supplementary material for: Engagement in local and collaborative wildfire risk mitigation planning across the western U.S.—Evaluating participation and diversity in Community Wildfire Protection Plans
Source: PLoS One. 2022 Feb 9;17(2):e0263757. doi: 10.1371/journal.pone.0263757 (PMC8827427; doi:10.1371/journal.pone.0263757)
Supplement: S1 File — (DOCX) [file pone.0263757.s001.docx]

Supporting Information for:

Engagement in local and collaborative wildfire risk mitigation planning across the western U.S. — Evaluating participation and diversity in Community Wildfire Protection Plans

Emily Palsa, Matt Bauer, Cody Evers, Matt Hamilton, Max Nielsen-Pincus

Correspondence to palsa.3@buckemail.osu.edu (EST)

**This Supporting Information includes:**

S0_1. Metadata for the plan-level dataset

S0_2. Metadata for the participant-level dataset

S0_3. Metadata for the planning boundaries dataset

S0_4. Metadata for the regression analysis dataset

S0_5. Regression analysis summary tables

**Other Supporting Information for this paper includes the following data and code:**

S1_plan_level.csv

S2_participant_level.csv

S3_planning_boundaries.zip

S4_regression_dataset.csv

S5_regression_code.R

**S0_1. Metadata for plan-level dataset**

The S1_plan_level.csv dataset was used in the creation of Table 1 and Figure 4 and contains a list of CWPPs used in this study with the attributes described below.

Table S0_1. Metadata associated with the plan-level dataset

| Attribute | Description |
| --- | --- |
| uid_year | 9-character code unique to each CWPP document.  Formatted: state abbreviation, random 3-digit number, publication year |
| cwpp_code | Code unique to each CWPP boundary.  Formatted: state abbreviation, shortened CWPP name, CWPP level |
| state | Abbreviation of state of CWPP publication |
| cwpp_level | CWPP spatial/administrative level. Includes:  FPRD = Fire protection district  CNTY = County or multiple counties  COMM = Communities (neighborhoods, towns, cities, etc.) |
| date | Date (year) of CWPP publication. |
| update | Binary indicator of if CWPP document is an original (0) or update of pre-existing plan (1) according to our date of publication. |
| prepared_by_consultants | Binary indicator of if CWPP was prepared by a private consultant (1) or not (0). |
| participants_in_cwpp | Binary indicator of if CWPP contained any instances of participation in its creation that we were able to identify (1) or not (0). |
| cwpp_jurisdiction_source | Source that we used for delineating the CWPP plan boundary. |

**S0_2. Metadata for participant-level dataset**

The S2_participant_level.csv dataset was used in creation of Table 1, Table 2, and Figure 5 and contains an anonymized list of instances of CWPP participation coded from CWPP documents with the attributes described below.

Table S0_2. Metadata associated with the participant-level dataset

| Attribute | Description |
| --- | --- |
| pid | Participation identification code. Randomly assigned and unique to each instance of participation. |
| uid_year | 9-character code unique to each CWPP document.  Formatted: state abbreviation, random 3-digit number, publication year |
| cwpp_code | Code unique to each CWPP boundary.  Formatted: state abbreviation, shortened CWPP name, CWPP level |
| high_level_affiliation | Category of participant's associated organization. Includes:  Federal = part of federal-level government State = part of state-level government Local = part of local-level government NGO = non-governmental organization  Unknown = unknown organizational affiliation |
| affiliation_code | Category of participant's associated organization. Includes:  BLM = unit/office of the Bureau of Land Management  City Organization = unit/office of a city-level government agency  County Organization = unit/office of county-level government agency  Community Fire Organization = Firewise organization, fire safe council  Homeowners Association = homeowners association  Individual = private individual (e.g., local resident)  Local Fire District or Department = local fire district or department  Non-Profit Organization = non-profit organization  Other Federal Agency = unit/office of a federal agency other than the USFS or BLM  Other Local or Regional Government Organization = unit/office of a government agency that is local/regional but not city or county  Other State Agency = unit/office of a state agency other than the State Dept of Forestry or Natural Resources agency  Private Business = private corporation  State Dept of Forestry or Natural Resources = unit/office of the state-level agency responsible for management of forests and/or natural resources  Tribal Organization = unit/office of a tribal government agency  University = unit of a university  Unknown = unknown organizational affiliation  USFS = unit/office of the U.S. Forest Service |
| pax_def_participated | Binary reflecting researchers’ confidence that the individual participated in the development of the CWPP (1) or not (0) as participation is defined in manuscript. Instances of "0" were excluded from analyses. |

**S0_3. Metadata for planning boundaries dataset**

Planning boundaries in the S3_planning_boundaries.zip dataset were either georeferenced directly from CWPP documents or were obtained from secondary sources if possible and were combined into a shapefile within QGIS. This dataset was used in the creation of Figure 3. The attributes of each boundary are described below.

Table S0_3: Metadata associated with the planning boundaries dataset

| Attribute | Description |
| --- | --- |
| UID | 5-character code unique to each CWPP boundary.  Formatted: state abbreviation, random 3-digit number |
| cwpp_code | Code unique to each CWPP boundary.  Formatted: state abbreviation, shortened CWPP name, CWPP level |

**S0_4. Metadata for regression analysis dataset**

Our model estimates the effects of a range of factors—related to the CWPP planning process, the planning context, and the broader socio-environmental context —on participation and diversity in the CWPP planning process. The S4_regression_dataset.csv was used in the creation of Table 4. The attributes of this dataset are described below.

Table S0_4. Metadata associated with the regression analysis dataset

| Attribute | Description |
| --- | --- |
| uid_year | 9-character code unique to each CWPP document.  Formatted: state abbreviation, random 3-digit number, publication year |
| participation | Participant count, scaled from 0-1. |
| diversity | Participant diversity calculated with the Shannon-Wiener Diversity Index, scaled from 0-1. |
| state | Abbreviation of state in which the CWPP was developed |
| cwpp_level | CWPP spatial/administrative level. Includes:  FPRD = Fire protection district  CNTY = County or multiple counties  COMM = Communities (neighborhoods, towns, cities, etc.) |
| year_range | Period in which CWPP was developed. Includes:  Year published: 2001-2007  Year published: 2008-2014  Year published: 2015-2021 |
| revision | Binary indicator of if CWPP document is an original (0) or update of pre-existing plan (1) according to our date of publication. |
| prepared_by_consultants | Binary indicator of if CWPP was prepared by a private consultant (1) or not (0). |
| population | Estimated population of planning area derived from the 2019 American Community Survey (Total Population variable), scaled from 0-1. |
| urban_rural_gradient | Proportion of population living in urban areas, based on data obtained from the 2010 U.S. Census (Urban variable). Urban population was divided by total population. |
| land_ownership_diversity | Data on land ownership were obtained from the USGS Protected Areas Database (USGS 2016), which we grouped into 14 major land classes. Using the proportions of areas in these classes we used the Shannon-Wiener index to calculate diversity of land ownership, which we subsequently rescaled from 0-1. |
| planning_area | Area encompassed by the CWPP planning boundary, scaled from 0-1. |
| structural_density | Density of housing units (number of units divided by planning area), using data on housing units obtained from the 2019 American Community Survey. Variable scaled from 0-1. |
| wildfire_risk | Data on wildfire risk were derived from the Wildfire Hazard Potential WHP dataset (Dillon et al. 2015), which assigns pixels a ranking based on the potential for fires that would be difficult to suppress. Pixel values within CWPP planning areas were averaged. Variable scaled from from 0-1. |
| social_vulner_index | CDC/ATSDR Social Vulnerability Index, scaled from 0-1. |

**S0_5. Regression analysis summary tables**

Table S0_5 reports summary statistics for the mixed-effects random-intercept model depicted in Table 4.

Participation model

|  | Estimate | Std. Error | df | t value | Pr(>\|t\|) |
| --- | --- | --- | --- | --- | --- |
| (Intercept) | 0.08 | 0.02 | 909.49 | 5.06 | 0.00 |
| priv_cons.rescaled | 0.01 | 0.01 | 971.31 | 1.07 | 0.28 |
| cwpp_levelCOMM | (0.03) | 0.01 | 852.30 | (3.04) | 0.00 |
| cwpp_levelFPRD | (0.03) | 0.01 | 898.55 | (2.64) | 0.01 |
| year_rangelate | 0.02 | 0.01 | 989.91 | 1.89 | 0.06 |
| year_rangemid | 0.01 | 0.01 | 989.97 | 0.87 | 0.39 |
| revision | (0.03) | 0.01 | 928.60 | (3.82) | 0.00 |
| sq_km.rescaled | 0.10 | 0.04 | 756.95 | 2.65 | 0.01 |
| state1_AZ | 0.00 | 0.01 | 761.78 | 0.20 | 0.84 |
| state2_CA | 0.01 | 0.01 | 787.18 | 1.86 | 0.06 |
| state3_CO | (0.00) | 0.01 | 943.62 | (0.44) | 0.66 |
| state4_ID | 0.05 | 0.01 | 536.87 | 4.53 | 0.00 |
| state5_MT | (0.02) | 0.01 | 969.89 | (1.71) | 0.09 |
| state6_NM | 0.01 | 0.01 | 951.81 | 0.78 | 0.44 |
| state7_NV | (0.01) | 0.02 | 593.97 | (0.86) | 0.39 |
| state8_OR | (0.00) | 0.01 | 877.82 | (0.08) | 0.93 |
| state9_UT | (0.03) | 0.01 | 959.94 | (2.46) | 0.01 |
| state10_WY | 0.03 | 0.01 | 966.18 | 3.17 | 0.00 |
| population.rescaled | 0.03 | 0.04 | 587.65 | 0.74 | 0.46 |
| urban_pop.rescaled | 0.00 | 0.01 | 859.41 | 0.03 | 0.97 |
| ownswdi.rescaled | 0.03 | 0.02 | 882.51 | 2.28 | 0.02 |
| wildfire.rescaled | (0.00) | 0.02 | 935.50 | (0.07) | 0.94 |
| houses_km.rescaled | (0.04) | 0.04 | 974.71 | (0.98) | 0.33 |
| total_social_vuln.rescaled | (0.02) | 0.01 | 876.41 | (1.11) | 0.27 |

Diversity model

|  | Estimate | Std. Error | df | t value | Pr(>\|t\|) |
| --- | --- | --- | --- | --- | --- |
| (Intercept) | 0.44 | 0.04 | 869.79 | 10.78 | 0.00 |
| priv_cons.rescaled | 0.79 | 0.08 | 976.45 | 10.25 | 0.00 |
| cwpp_levelCOMM | 0.01 | 0.01 | 971.87 | 0.85 | 0.39 |
| cwpp_levelFPRD | (0.04) | 0.02 | 793.04 | (1.67) | 0.09 |
| year_rangelate | (0.02) | 0.03 | 845.53 | (0.70) | 0.48 |
| year_rangemid | 0.00 | 0.02 | 987.85 | 0.05 | 0.96 |
| revision | 0.03 | 0.02 | 987.37 | 1.78 | 0.08 |
| sq_km.rescaled | 0.03 | 0.02 | 808.77 | 1.45 | 0.15 |
| state1_AZ | (0.02) | 0.09 | 688.26 | (0.24) | 0.81 |
| state2_CA | 0.06 | 0.03 | 698.63 | 1.91 | 0.06 |
| state3_CO | 0.03 | 0.02 | 720.47 | 1.37 | 0.17 |
| state4_ID | 0.01 | 0.02 | 902.20 | 0.33 | 0.74 |
| state5_MT | (0.08) | 0.03 | 469.12 | (2.99) | 0.00 |
| state6_NM | (0.07) | 0.03 | 939.91 | (2.22) | 0.03 |
| state7_NV | 0.04 | 0.03 | 909.19 | 1.41 | 0.16 |
| state8_OR | 0.03 | 0.04 | 485.49 | 0.85 | 0.39 |
| state9_UT | 0.02 | 0.03 | 820.47 | 0.97 | 0.33 |
| state10_WY | (0.11) | 0.03 | 925.63 | (3.73) | 0.00 |
| population.rescaled | 0.08 | 0.03 | 935.95 | 3.19 | 0.00 |
| urban_pop.rescaled | (0.06) | 0.10 | 496.36 | (0.67) | 0.50 |
| ownswdi.rescaled | (0.01) | 0.02 | 797.09 | (0.38) | 0.71 |
| wildfire.rescaled | 0.15 | 0.04 | 825.19 | 3.84 | 0.00 |
| houses_km.rescaled | 0.02 | 0.04 | 892.16 | 0.36 | 0.72 |
| total_social_vuln.rescaled | (0.03) | 0.10 | 947.08 | (0.35) | 0.72 |

**S0_6. Correlation table**

**
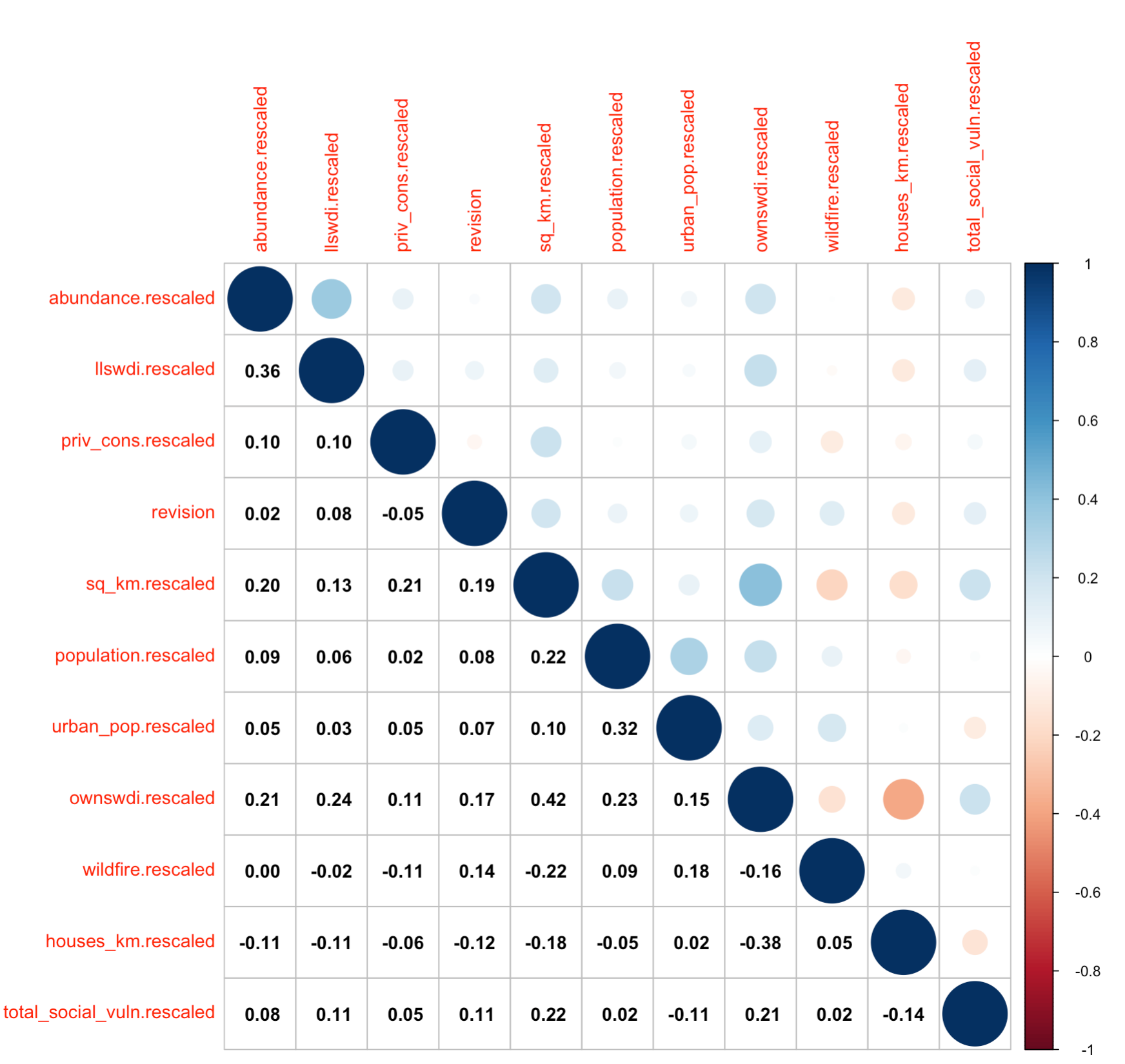
**

References:

Dillon, G.K., Menakis, J., Fay, F., 2015. Wildland Fire Potential: A Tool for Assessing Wildfire Risk and Fuels Management Needs, in: Keane, R.E., Jolly, W.M., Parsons, R., Riley, K. (Eds.), Proceedings of the Large Wildland Fires Conference; May 19-23, 2014; Missoula, MT. Proc. RMRS-P-73. U.S. Department of Agriculture, Forest Service, Rocky Mountain Research Station, Fort Collins, CO, pp. 60–76.

USGS, 2016. Protected Areas Database of the United States (PAD-US), version 1.4 Combined Feature Class, US Geological Survey, Virginia, USA.

US Census Bureau, 2001a. Census 2000 Summary File 1. U.S. Census Bureau, Washington, DC.

ACS
